# Research on cotton plant type identification method based on multidimensional vision

**DOI:** 10.3389/fpls.2025.1610577

**Published:** 2025-10-13

**Authors:** Ying Liu, Bo Liu, Weihua Fu, Jiajie Yang, Xiaotong Zheng, Xiantao Ai, Xiaojuan Li

**Affiliations:** ^1^ School of Intelligent Manufacturing Modern Industry, Xinjiang University, Urumqi, China; ^2^ Western Agricultural Research Center, Chinese Academy of Agricultural Sciences, Changji, China; ^3^ School of Wisdom Agriculture, Xinjiang University, Urumqi, China

**Keywords:** three-dimensional reconstruction, two-dimensional projection, fast convex hull, corner change rate, plant type

## Abstract

**Introduction:**

Plant type is an important part of plant phenotypic research, which is of great significance for practical applications such as plant genomics and cultivation knowledge modeling. The existing plant type judgment mainly relies on subjective experience, and lacks automatic analysis and identification methods, which seriously restricts the progress of efficient crop breeding and precision cultivation.

**Methods:**

In this study, the digital structure model of cotton plant was constructed based on multi-dimensional vision, and the rapid analysis and identification method of cotton plant type was established. 50 cotton plants were used as experimental objects in this study. Firstly, multi-view images of cotton plants at boll opening stage were collected, and a three-dimensional point cloud model of cotton plants was constructed based on Structure From Motion and Multi View Stereo (SFM-MVS) algorithm. The original cotton point cloud data was preprocessed by coordinate correction, statistical filtering, conditional filtering and down-sampling to obtain a high-quality three-dimensional model. The three-dimensional model is projected in two dimensions to obtain the two-dimensional projection data of cotton plants from multiple perspectives. Secondly, based on the fast convex hull algorithm, the cotton plant two-dimensional convex hull was constructed from multiple perspectives, and the distribution range and corner change rate of each corners of the convex hull were analyzed, and the identification basis of cotton plant type was established.

**Results:**

The R2 of plant height and width extracted from the model were greater than 0.90, and RMES were 0.372 cm and 0.387 cm, respectively. When the maximum number of point clouds is 75335, the point cloud reading time, cotton multi-view projection time, and convex hull automatic construction time are 0.402 S, 2.275 S, and 0.018 S, respectively. Finally, the cotton cylinder type classification interval is 0-0.2, and the tower type classification interval is 0.4-1.5.

**Discussion:**

The cotton plant type identification method proposed in this study is fast and efficient. It provides a solid theoretical basis and technical support for cotton plant type identification.

## 1 Introduction

Cotton is a valuable economic crop and the main raw material of the global textile Industry (Dai et al., 2017; Li et al., 2021). In 2023, the cotton planting area in Xinjiang was 2369.3 khm^2^, with a total output of 5.112 million tons, accounting for 91% of the national total output. Plant type is a key factor affecting cotton yield, early maturity and mechanized harvesting, and is an indispensable part of crop breeding. The ideal plant type is helpful to increase planting density, improve photosynthetic efficiency and reduce yield loss during mechanized harvesting (Wang et al., 2024). Cotton plant type is mainly divided into cylinder type and tower type (Du and Zhou, 2005). At present, cotton plant type is mainly judged by visual observation, lacking quantitative evaluation system and standard. It is urgent to construct a rapid, accurate and undamaged cotton plant type analysis and identification method.

With the rise of smart agriculture, three-dimensional reconstruction technology has become an important means to capture shape and structure information (Yu et al., 2024a) (Yang et al., 2024)used the improved Structure From Motion algorithm to achieve high-precision three-dimensional reconstruction and trait measurement of complex plants. The R^2^ of plant height and plant width was above 0.999 (Sha et al., 2025b)used a variety of optimization strategies to perform three-dimensional reconstruction of transparent objects under limited constraints. The error indexes CD and CDN-mean were 1.81 and 5.62, respectively. The results show that the proposed TransNeXt achieved better results (Zou et al., 2024)used TOF sensor to capture the three-dimensional geometric structure of fruit trees, used Delaunay triangulation algorithm and Dijkstra shortest path algorithm to calculate the minimum spanning tree, and completed the construction of high-precision fruit tree point cloud model. The accuracy deviation between the constructed three-dimensional point cloud and skeleton model of fruit trees and the measured data is kept within 7% (Wu et al., 2024)used the MVS-Pheno high-throughput phenotypic platform to obtain high-precision three-dimensional point clouds of wheat plants, and then based on the SoftGroup network model, the point cloud organ segmentation and morphological parameter extraction of wheat plants were performed. The accuracy of organ semantic segmentation was 95.2%, and R^2^ of leaf length and width was above 0.80 (Wu et al., 2019)accurately extracted maize plant skeleton based on three-dimensional point cloud, and used the extracted plant skeleton to estimate morphological parameters such as leaf inclination angle and leaf growth height. The extracted phenotypic parameters R^2^ were all above 0.93. At the same time, the method based on agricultural big model has strong application potential in the fields of crop classification and recognition (Guo et al., 2024) (Gurav et al., 2023)proposed to use SAM to divide the field contour of satellite images as the basis for crop classification, and used Clustering Consensus Metrics to evaluate its ability, as the basis of subsequent crop classification and map generation process (Tan et al., 2023)designed an experiment to identify farmland crops based on remote sensing images and corresponding basic information for GPT-4. The results show that GPT-4 performs well on general images (Li et al., 2023b)used SAM adapter for image segmentation of pests and diseases, especially in the identification of coffee leaf diseases, the average Dice coefficient and the average cross-over score increased by about 40%. Based on prediction models, deep learning, unsupervised training and other methods, the application of large models has been promoted (Alhatemi and Savaş, 2024; Fei et al., 2025; Liu et al., 2025; Sha et al., 2025c; Xiang et al., 2025). In summary, the technical methods based on three-dimensional reconstruction and large-scale agricultural models have become an important method for studying plant phenotypes, and are the primary prerequisites for crop identification and classification and phenotypic data analysis.

The construction of three-dimensional model of plants based on three-dimensional reconstruction technology has become an important method to study the phenotypic structure of plants, and is the primary prerequisite for the analysis of crop phenotypic data.

Plant type research is attracting widespread attention (Zhao et al., 2024b)reviewed the morphological characteristics of crop ideal plant type from four aspects: leaf, stem, panicle and root, and summarized the cultivation techniques of ideal plant type regulation, providing a theoretical basis for the cultivation of ideal plant type (Li et al., 2023a)proposed four stages of ideal plant type of rapeseed, constructed the index system of ideal plant type of rapeseed, and discussed the basic characteristics, construction strategies and research trends of ideal plant type of rapeseed. At present, most of the plant type research stays at the level of review and qualitative analysis. There have been many reports on wheat, rice, corn, flue-cured tobacco and other crops, but there are relatively few studies on cotton. The research on automatic analysis and identification of cotton plant type based on three-dimensional model is limited.

**Figure 1:**
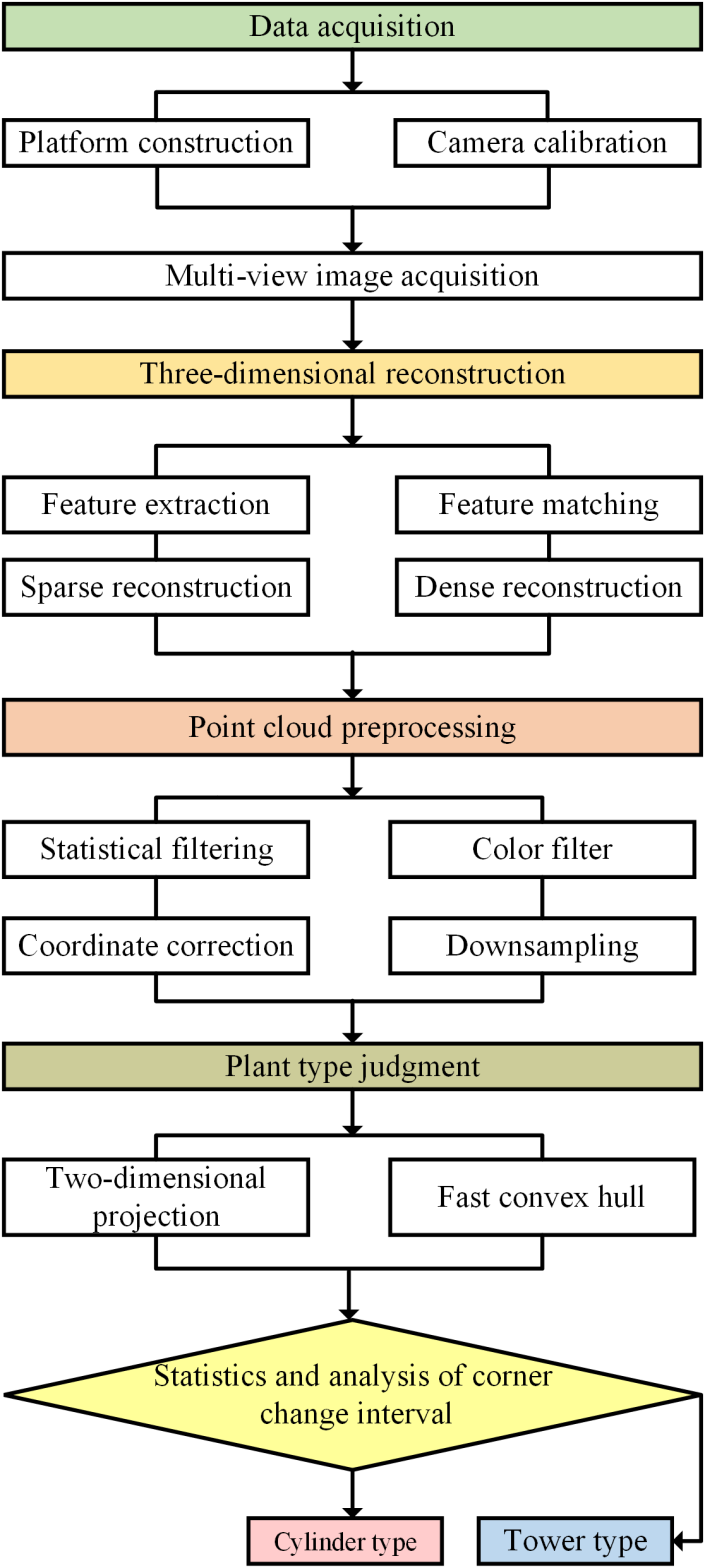
Main process of this study.

The plant type of cotton is a complex trait, which is controlled by genotype and environment. There is a correlation between plant type traits and yield, quality and early maturity traits. Plant type breeding is an effective way to improve cotton yield and fiber quality (Fu et al., 2019). Quantitative analysis of cotton plant type was carried out to improve the identification accuracy of cotton plant traits and promote the application of plant type research in breeding. In summary, based on the above research, this study proposes a cotton plant type identification method based on multi-dimensional vision with cotton as the research object. The main work is as follows:

1. The three-dimensional model of multiple cotton plants was obtained by using the low-cost cotton three-dimensional reconstruction method based on Structure From Motion and Multi View Stereo (SFM-MVS) algorithm.
2. The three-dimensional point cloud model of cotton is preprocessed, and the point cloud coordinates of cotton are corrected by rotation and translation matrix to make it consistent with the growth direction. Statistical filtering and color-based conditional filtering are used to denoise the point cloud to obtain a pure point cloud model. Then, the number of three-dimensional model point clouds is reduced by down-sampling, and the running speed of the algorithm is improved.
3. A low-cost cotton plant type determination method based on multi-view two-dimensional projection and fast convex hull algorithm was proposed. The main process of this study is shown in **Figure 1**.

## 2 Materials and methods

### 2.1 Plant materials

**Figure 2:**
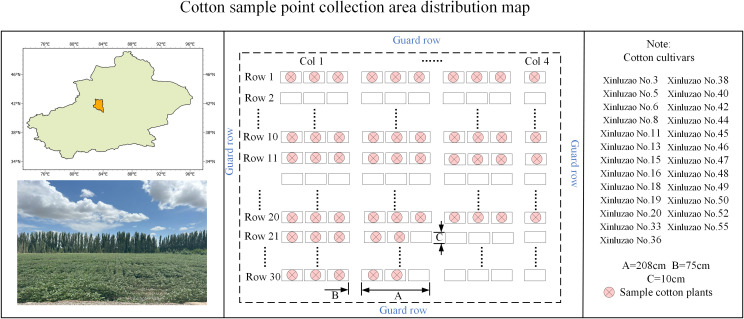
Experimental field distribution map.

Taking cotton as the experimental object of this study, from April to November 2024, data collection was carried out at the cotton base of Kuche Modern Agricultural Science and Technology Innovation Center. A total of 25 varieties were selected, 2 plants for each variety, and a total of 50 cotton plant samples were selected. Among them, 20 cotton plants were used as validation set. The field distribution position is shown in **Figure 2**, and the cotton plants were moved to the flowerpot and moved indoors.

### 2.2 Data acquisition

**Figure 3:**
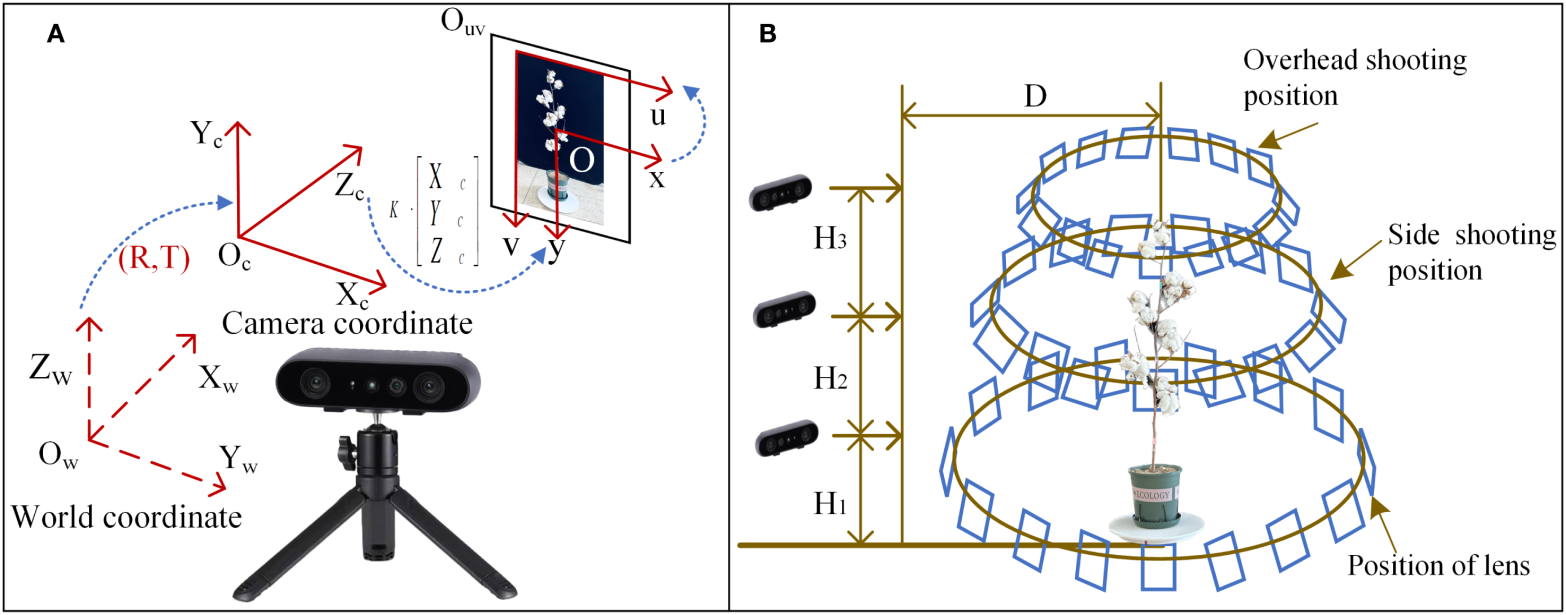
**(A)** Data acquisition principle. **(B)** Data acquisition scheme.

In order to obtain clear and high-quality data, an indoor image acquisition platform composed of a tripod, a binocular structured light camera, a black screen, a 39 cm-diameter electric turntable and a computer is built. In this study, ORBBEC Gemini 2 is used for data shooting. The intrinsic parameters of the camera are shown in **Table 1**. The depth range of the camera is 0.2 m-5 m. This camera uses a depth sensing system, which uses active infrared (IR) stereo vision and inertial measurement unit (IMU). The system uses structured light technology to work through a pattern projector to create the difference between the stereo images captured by two infrared cameras. Accurate direction parameters are provided by 6-axis IMU. The Lenovo computer is used for later data processing and analysis. The computer is configured as: Windows 64-bit operating system, 32G running memory, inter (R) Core (TM) i7-14700HX CPU, RTX 4060 graphics card. The principle of coordinate transformation between cameras is shown in **Figure 3A**. The camera calibration process is as follows:

1. Prepare a chessboard with a grid size of 15 mm * 15 mm, and calibrate the camera by Zhang Zhengyou calibration method.
2. Detect the corner points on the chessboard and obtain their pixel coordinates;
3. Determine the internal parameter matrix $\text{K}=[\begin{array}{ccc} 456.1847 & 0 & 683.9744\\ 0 & 457.5567 & 336.5738\\ 0 & 0 & 1 \end{array}]$ , The distortion coefficient ${\text{k}}_{1}=0.0027$ , ${\text{k}}_{2}=-0.0041$ .

**Table 1:** Camera specifications.

Parameter	Value
Image width (pixels)	1280
Image weight (pixels)	800
Visual angle (°)	91*66
Frame rate (fps)	30

Mean Reprojection Error = 0.25 pixel. Reprojection error is small, indicating the accuracy of the calibration results.

In the process of camera calibration, slight blurring and ghosting of the image may occur, resulting in excessive differences in multiple repeated calibration results. In order to reduce the image shooting error, this manuscript increases the camera bracket to more accurately control the camera ‘s angle and height, and improve the calibration accuracy.

The camera faces the cotton plant directly and locates it accurately. The fixed horizontal distance between the camera and the center of the turntable is 2.23 m. The turntable rotates at a fixed speed of 10 S to shoot cotton images. and a total of 249 high-resolution images are obtained. Considering that the growth height of cotton can reach 1.2m-1.5m and the morphological structure is complex, this study adopts three different heights to rotate clockwise to shoot cotton plants. The data acquisition scheme is shown in **Figure 3B**. The shooting distance D and the ground height H of the camera from the cotton plant are measured by a laser rangefinder. The plant height and plant width of training set were measured with a ruler, and the mean values were measured 7 times and recorded in **Table 2** as the data source for subsequent correlation analysis.

**Table 2:** Manual measurement data.

Cotton varieties	Average plant height/cm	Average plant width/cm	Cotton varieties	Average plant height/cm	Average plant width/cm
Xinluzao No.3	96.4	35.2	Xinluzao No.13	104.7	88.7
	126.5	144.1		80.2	32.6
Xinluzao No.5	93.4	38.1	Xinluzao No.15	77.5	29.4
	73.5	32.3		89.7	34.2
Xinluzao No.6	72.3	28.6	Xinluzao No.16	89.9	55.7
	71.5	30.2		85.9	46.2
Xinluzao No.8	88.8	33.9	Xinluzao No.18	94.4	71.1
	97.2	43.5		93.4	41.2
Xinluzao No.11	108.4	82.9	Xinluzao No.19	79.4	28.9
	101.6	89.4		111.6	58.6
Xinluzao No.20	78.2	47.8	Xinluzao No.33	108.5	70.6
	110.5	68.6		97.4	35.8
Xinluzao No.36	88.6	56.7	Xinluzao No.38	76.8	37.6
	109.7	43.5		89.9	56.7
Xinluzao No.55	95.2	39.5			
	77.5	34.3			

### 2.3 Three-dimensional reconstruction and data preprocessing method

#### 2.3.1 Three-dimensional reconstruction method of cotton plant

**Figure 4:**
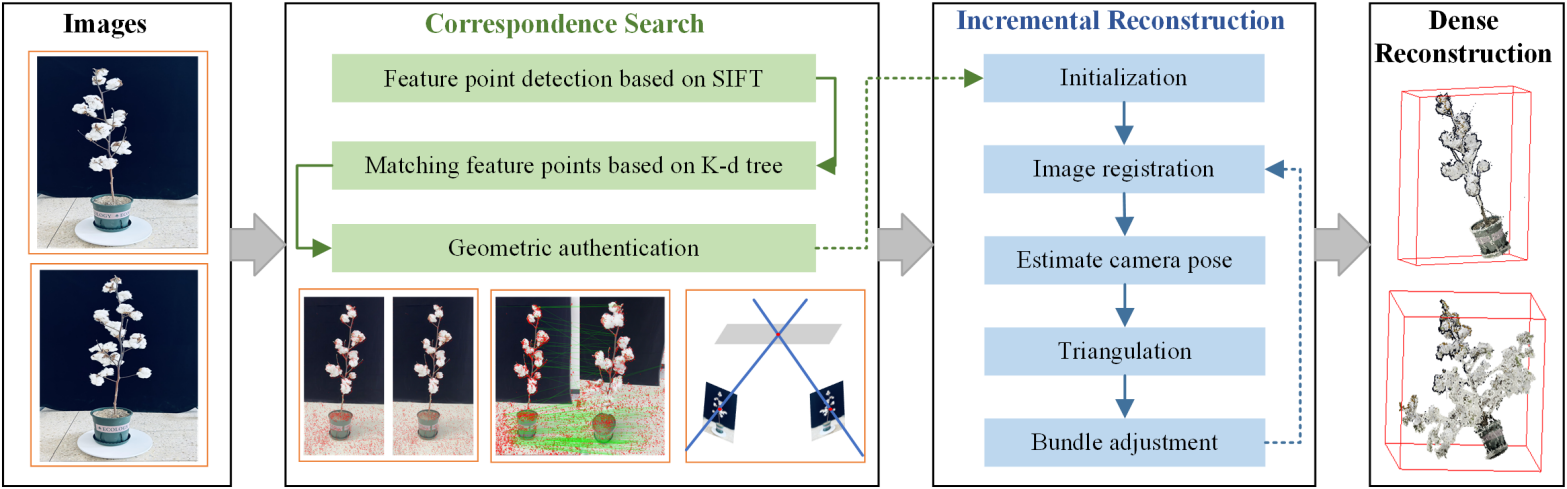
The pipeline of three-dimensional reconstruction.

The process of three-dimensional reconstruction method is shown in **Figure 4**. Firstly, the image acquisition platform is used to capture the multi-temporal plant images of cotton. Secondly, the three-dimensional point cloud model of cotton is reconstructed by using the SFM-MVS algorithm. Finally, the point cloud model is subjected to coordinate correction, filtering and down-sampling operations to obtain a pure point cloud model.

The core of the algorithm mainly includes feature point extraction, stereo matching, pose estimation and parameter optimization (Schönberger and Frahm, 2016; Schönberger et al., 2016).The specific reconstruction process is as follows:

(1) Feature point extraction and matching: A set of cotton sequence images are input, and the feature points in multi-view two-dimensional images are detected and extracted by Scale-Invariant Feature Transform (SIFT). The K-dimensional tree is used to calculate the Euclidean distance between the feature points of the two images for stereo matching of the feature points. Finally, the projection geometry is used to map the transformation of the feature points between the images to verify the matching.

(2) Incremental sparse point cloud reconstruction: Based on the correspondence between two-dimensional - three-dimensional, the triangulation is used to expand the point set and estimate the spatial pose of the camera, and then the Bundle Adjustment (BA) method is used to iteratively optimize the minimum reprojection error E of the Equation 1. Finally, the sparse point cloud reconstruction of cotton is realized.


(1) $$\begin{array}{c} E=\sum_{j}\rho_{j}(\pi {\left(P_{c},X_{k}\right)}^{2}) \end{array}$$

P_c_ is the camera parameter, X_k_ is the point parameter, ${\text{ρ}}_{\text{j}}$ is the loss function, x_j_∈R^2^ represents the image coordinates of the feature points.

(3) Dense point cloud reconstruction: In the process of dense reconstruction, due to the distorted image, there will be a large disparity estimation error at the edge. Firstly, the multi-view plant image is de-distorted. Combined with the optical consistency and geometric consistency in multi-view, the depth map and normal vector map of multi-view are estimated and optimized. Finally, the depth map fusion is used to reconstruct the dense three-dimensional point cloud of cotton.

#### 2.3.2 Point cloud coordinate correction

In order to accurately obtain the cotton plant type information, it is necessary to correct the coordinates of the three-dimensional point cloud of cotton (Huang et al., 2019). Because there is a certain deviation between the main direction of the reconstructed cotton three-dimensional point cloud model and the growth direction of the real cotton plant, it is necessary to use the translation and rotation matrix for coordinate transformation, so that the main direction of the three-dimensional point cloud of the cotton plant is consistent with the growth direction. The coordinate transformation is shown in Equation 2. T_A_ is the three-dimensional point cloud of cotton after coordinate transformation, M_T_ is the translation and rotation matrix, T_O_ is the original three-dimensional point cloud of cotton plant.


(2) $$\begin{array}{c} T_{A}=M_{T}T_{O} \end{array}$$

#### 2.3.3 Point cloud denoising

Due to the influence of equipment accuracy and environmental factors, there are two kinds of noise in the reconstructed point cloud. One is the outliers scattered throughout the plant, which are removed by statistical filtering. The other is black noise uniformly distributed along the edges of cotton stems and leaves, which is removed by color-based conditional filtering. The non-target plants were cut based on the CloudCompare software before removing the noise.

(1) Statistical filtering.

For cotton point cloud data, the average distance from each point to its nearest k points is calculated, and the neighborhood of each point is statistically analyzed (Han et al., 2017). The distance of all points in the point cloud constitutes a Gaussian distribution, where the mean *μ* and the standard deviation *σ* are determined by Equation 3. The coordinates of the nth point P_n_(X_n_,Y_n_,Z_n_) to any point P_m_(X_m_,Y_m_,Z_m_) is calculated by Equation 4.


(3) $$\begin{array}{c} \mu =\frac{1}{n}\sum_{i=1}^{n}S_{i},\sigma =\sqrt{\frac{1}{n}\sum_{i=1}^{n}{\left(S_{i}-\mu \right)}^{2}} \end{array}$$

(4) $$\begin{array}{c} S_{i}=\sqrt{{\left(X_{n}-X_{m}\right)}^{2}+{\left(Y_{n}-Y_{m}\right)}^{2}+{\left(Z_{n}-Z_{m}\right)}^{2}} \end{array}$$

The average distance between a point and its nearest k points is calculated. If the average distance is within the standard range (μ-σ•std, μ+σ•std*)*, the point is retained. Otherwise, it is judged as an outlier and removed, where std is the standard deviation multiple.

(2) Color-based conditional filtering.

The first step is to define a conditional filter based on the color attribute of the point (Watanabe et al., 2023), and create a multi-condition composite conditional object, which is composed of RGB color components.

The second step: create a condition to remove the filter object, input the condition object and the point cloud to the filter. The color component threshold is set separately, each point in the input point cloud is traversed, and compared with the color threshold. If all conditions are met, the point is retained, otherwise the point is discarded, and the operation is repeated. Finally, the qualified points are stored in the new point cloud object.

#### 2.3.4 Point cloud down-sampling

The three-dimensional point cloud data of the original cotton plant is dense. In order to save the running time of the later algorithm, the voxel grid down-sampling method is used to simplify the cotton point cloud data. Point cloud down-sampling is to reduce the number of point clouds without changing the basic geometric characteristics of point clouds. A minimum three-dimensional voxel grid is created for the input point cloud data, and then the three-dimensional voxel grid is divided into several small grids. The point cloud data is placed in the corresponding small grid, and all the data points in the small cube grid are replaced by the voxel center of gravity to realize the point cloud down-sampling. The voxel grid down-sampling can well preserve the geometric features of the point cloud and prevent the loss of feature information (Lyu et al., 2024).The number of points after down-sampling is reduced to about 25% of the original point cloud, and the contour of the cotton plant is almost unchanged, which can improve the running speed of the algorithm and reduce the running time.

### 2.4 Identification method of cotton plant type

Reasonable plant type is conducive to improving planting density and photosynthetic efficiency. Suitable plant type structure is the key to mechanized harvesting of cotton. Cultivating and screening cotton varieties suitable for mechanical harvesting is an urgent problem to be solved in cotton production (Song et al., 2024).

#### 2.4.1 Fast convex hull algorithm of cotton plant

A convex hull is the smallest convex polygon or polyhedron that can contain all given points, which can determine the minimum enclosing shape of the point set (Zhou et al., 2024). The fast convex hull algorithm can construct the minimum bounding shape of the two-dimensional point set of cotton. By analyzing the minimum bounding shape of cotton, the geometric structure information of cotton can be obtained.

At present, the most commonly used convex hull algorithm is Andrew and Graham scanning method (Ma et al., 2017).However, both algorithms need to globally sort the contour point set based on the polar angle. When facing large-scale point set data, it takes a long time. Therefore, this study uses a fast convex hull algorithm that can save the time of solving the contour convex hull (Kirkpatrick and Seidel, 1986). It constructs the convex hull of cotton two-dimensional point set quickly and automatically by recursive divide-and-conquer method. Unlike other convex hull algorithms, the fast convex hull algorithm gradually constructs the convex hull by selecting the farthest point, without relying on global sorting. The specific steps are as follows:

(1) Select the reference points in the two-dimensional projection data of cotton and segment the point set. Traverse all the points in the point set, find the leftmost point and the rightmost point in the point set according to the coordinates, and mark them as A and B. A and B must be part of the convex hull, connect AB to form a datum line segment, and the datum line segment divides the cotton two-dimensional point set into upper and lower planes. Given the coordinates of the two ends of the reference line AB in the plane (X_A_,Y_A_), (X_B_,Y_B_) and any point P(X_P_,Y_P_), determine the positional relationship between point P and the straight line AB. The equation of straight line AB is as follows (5):


(5) $$\begin{array}{c} Y-Y_{A}=\frac{Y_{B}-Y_{A}}{X_{B}-X_{A}}(X-X_{A}) \end{array}$$

By substituting the point *P* into the Equation 5, the position relation *F* between the point *P* and the straight line AB is obtained as Equation 6:


(6) $$\begin{array}{c} F=X_{A}(Y_{B}-Y_{P})-Y_{A}(X_{B}-X_{P})+(X_{B}Y_{P}-X_{P}Y_{B}) \end{array}$$

(2) Select the farthest point in the two-dimensional point set of cotton. The farthest point must be on the convex hull. Find out the farthest point of the reference line AB in the upper and lower planes, marked as C and D respectively, forming two triangles ABC and ABD. The formula for calculating the distance *d* between any point (X,Y) in the point set and the line segment AB is as Equation 7:


(7) $$\begin{array}{c} d=\frac{\left|ax+by=c\right|}{\sqrt{a^{2}+b^{2}}} \end{array}$$

where $a=Y_{B}-Y_{A}$ , $b=X_{A}-X_{B}$ , $c=X_{B}Y_{A}-X_{A}Y_{B}$ .

(3) Take the triangle ABC and ABD as new sub-problems respectively, find out the farthest point of the original contour relative to each edge of the triangle, get the new triangle and the new farthest point, repeat the process until all points are processed.

(4) All the contour points found are the vertices of the convex hull, and all the vertices are connected into a closed curve, which is the two-dimensional minimum convex hull of cotton.

#### 2.4.2 Identification method of cotton plant type

There are two common cotton plant types: the upper and lower parts of the cylinder type are basically the same, and the lower part of the tower type is larger and the upper part is smaller. The existing cotton plant type identification methods are subjective, so this study proposes the plant type identification method based on the pre-processed cotton plant three-dimensional model.

**Figure 5:**
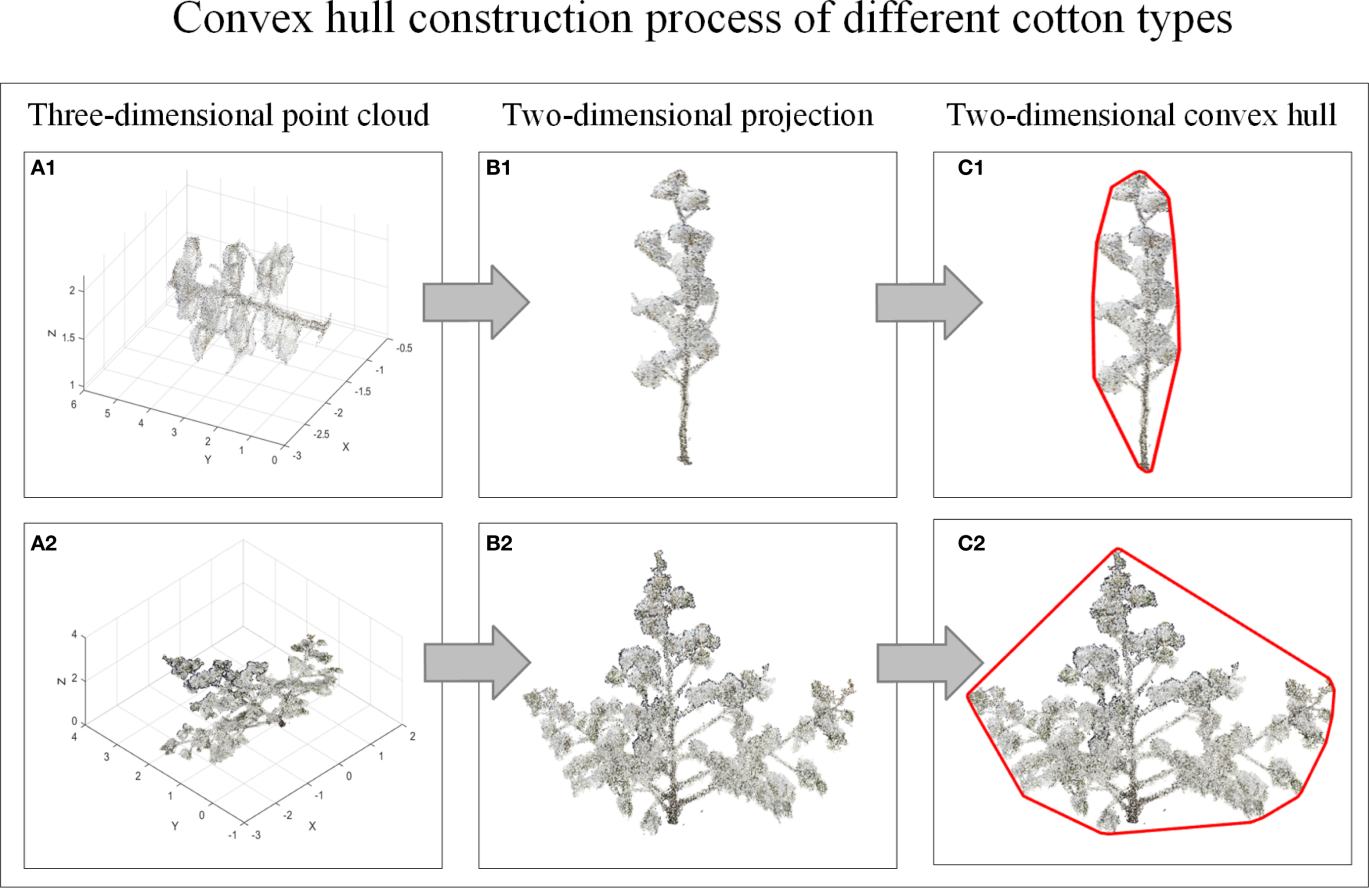
Flow chart of cotton plant type identification method. **(A1, A2)** Three-dimensional point cloud. **(B1, B2)** Two-dimensional projection. **(C1, C2)** Two-dimensional convex hull.

**Figure 6:**
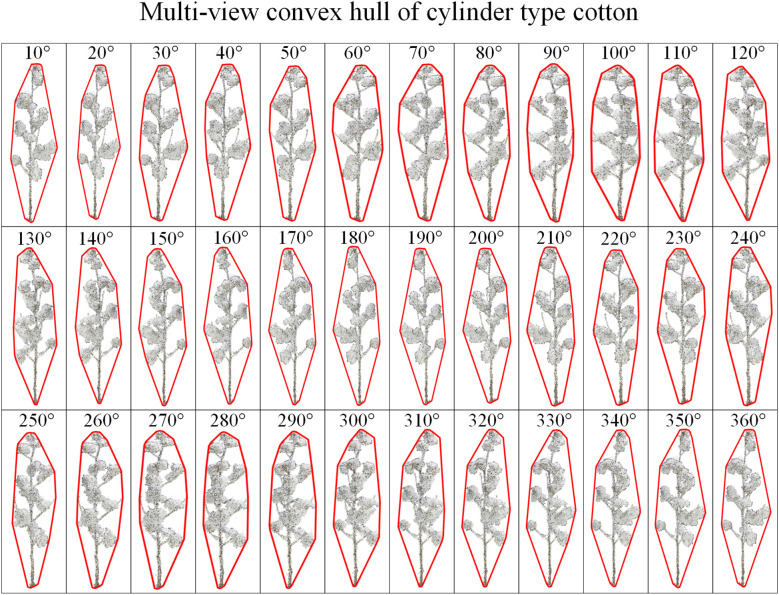
A cotton multi-view two-dimensional convex hull.

Three-dimensional can characterize the growth characteristics of crops in detail, but the cotton plant types observed from different perspectives are different. In order to reduce the visual error, this study rotates the three-dimensional model of cotton around the Y-axis and projects it to the XOY two-dimensional plane every 10°with Matlab2022. A total of 36 two-dimensional data of cotton plants were obtained, and then the minimum convex hulls were constructed for the two-dimensional data of multi-view cotton plants. **Figure 5** is the process of cotton plant type identification method. **Figure 6** is a convex hull of 36 viewing angles of a cotton plant, and the corner change rate of the convex hull is calculated respectively. Finally, the corner change rate of the convex hull of 36 viewing angles of each cotton plant is statistically analyzed, and the cotton plant type is further judged.

After the construction of the two-dimensional convex hull of the cotton plant, the coordinates of the convex hull corner points will be output. **Table 3** shows the coordinates of the convex hull corner points output after the projection and construction of the convex hull when the rotation angle is 90°, and then the change rate of the convex hull corner points is calculated by Equation 8. After the corner change rate is solved, the height is taken as the vertical axis, and the corner change rate is taken as the horizontal axis, and the change interval range diagram is made.

**Table 3:** Two-dimensional convex hull corner coordinates of cotton when the rotation angle is 90°.

Convex hull	Convex hull left corner coordinates/cm	Convex hull right corner coordinates/cm
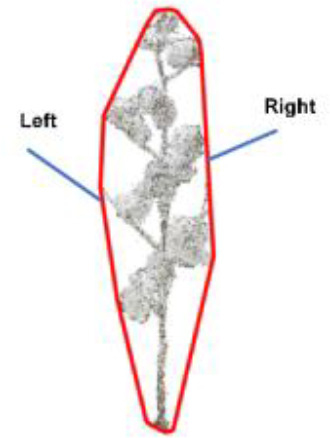	(5.47, 0.01)	(5.47, 0.01)
	(5.05, -0.26)	(4.95, 0.48)
	(4.22, -0.65)	(3.95, 0.57)
	(3.39, -0.69)	(3.07, 0.64)
	(2.05, -0.45)	(2.55, 0.62)
	(0.48, 0.04)	(0.48, 0.04)


(8) $$\begin{array}{c} K^{\prime }=\frac{\frac{Y_{3}-Y_{2}}{X_{3}-X_{2}}-\frac{Y_{2}-Y_{1}}{X_{2}-X_{1}}}{X_{3}-X_{1}} \end{array}$$

Where K’ is the corner change rate, (X_1_,Y_1_), (X_2_,Y_2_), (X_3_,Y_3_) are the corner coordinates of two-dimensional convex hull.

### 2.5 Evaluating indicator

1. Accuracy evaluation of reconstruction method.

Accuracy evaluation of reconstruction method: The correlation coefficient R^2^ and root mean square error (RMSE) between the measured values and the height and width values of cotton plants extracted from the reconstructed point cloud were calculated respectively to evaluate the error of reconstruction accuracy. RMSE is determined by Equation 9.


(9) $$\begin{array}{c} RMSE=\sqrt{\frac{1}{n}\sum_{i=1}^{n}{\left({\hat{y}}_{i}-y_{i}\right)}^{2}} \end{array}$$

where n is the number of samples, i is the current sample, ${\hat{y}}_{í}$ and ${\text{y}}_{\text{i}}$ are the predicted value and the true value, respectively.

2. Evaluation of plant type classification method.

(1) The Accuracy is expressed in Equation 10. It refers to the proportion of correct classification of plant type.


(10) $$\begin{array}{c} Accuracy=\frac{\text{TP}+\text{TN}}{\text{TP}+\text{FP}+\text{FN}+\text{TN}} \end{array}$$

(2) The Precision is expressed in Equation 11. It expressed the proportion of the predicted plant type to the true plant type.


(11) $$\begin{array}{c} Precision=\frac{\text{TP}}{\text{TP}+\text{FP}} \end{array}$$

(3) The Recall rate is expressed in Equation 12. It represents the proportion of plant type correctly predicted in the actual plant type.


(12) $$\begin{array}{c} Recall=\frac{\text{TP}}{\text{TP}+\text{FN}} \end{array}$$

## 3 Results

### 3.1 Point cloud visualization

**Figure 7:**
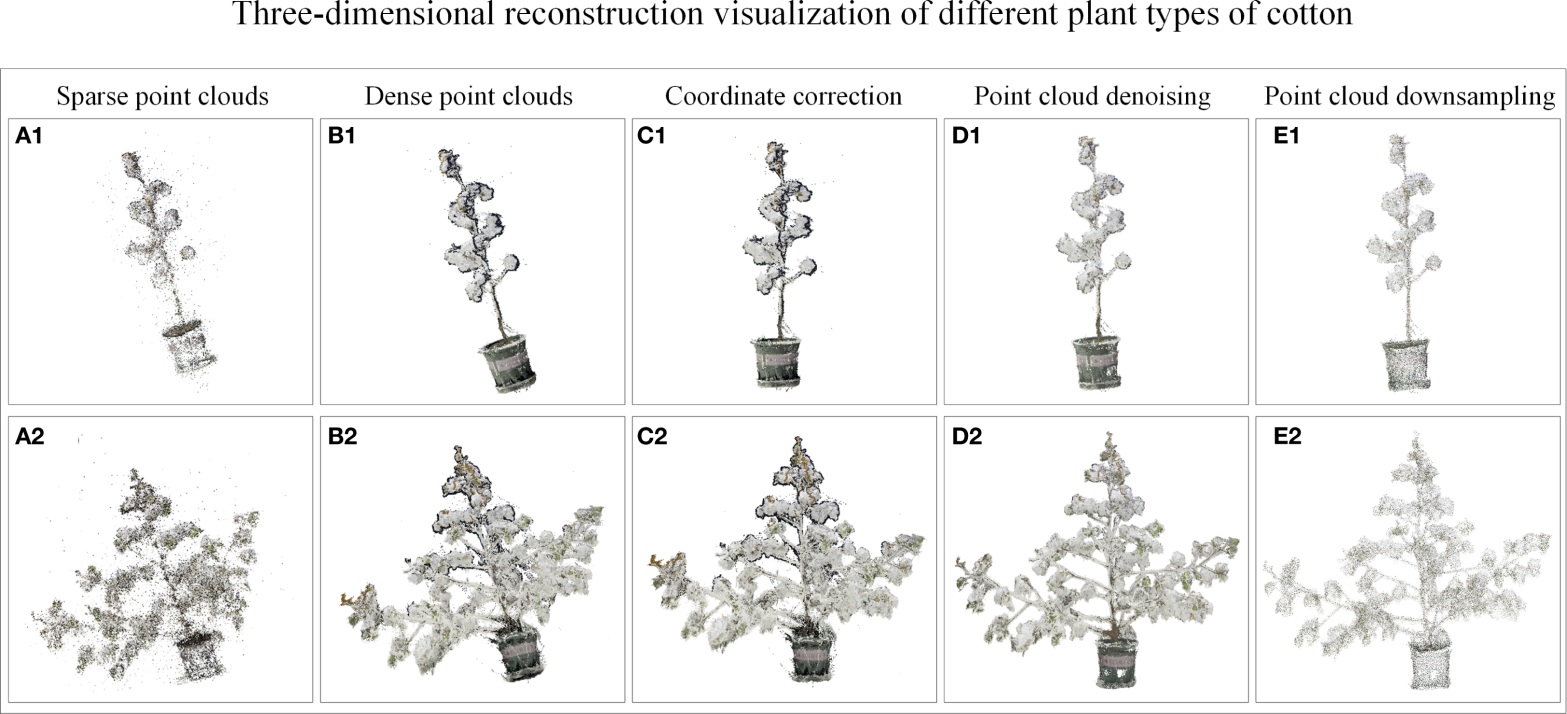
Point cloud visualization results of cylinder type and tower type cotton. **(A1, A2)** Sparse point clouds. **(B1, B2)** Dense point clouds. **(C1, C2)** Coordinate correction. **(D1, D2)** Point cloud denoising. **(E1, E2)** Point cloud down-sampling.

The three-dimensional reconstruction and denoising results of the point cloud are shown in **Figure 7**. Taking the varieties of Xinluzao No.11 and 15 as examples, the visualization results of the point cloud show that the algorithm can obtain the point cloud with clear structure and realistic color. The denoising method successfully eliminates the black noise of the leaf edge and the outliers around the plant, while retaining the three-dimensional information of the plant.

### 3.2 Accuracy of three-dimensional reconstruction algorithm

**Figure 8:**
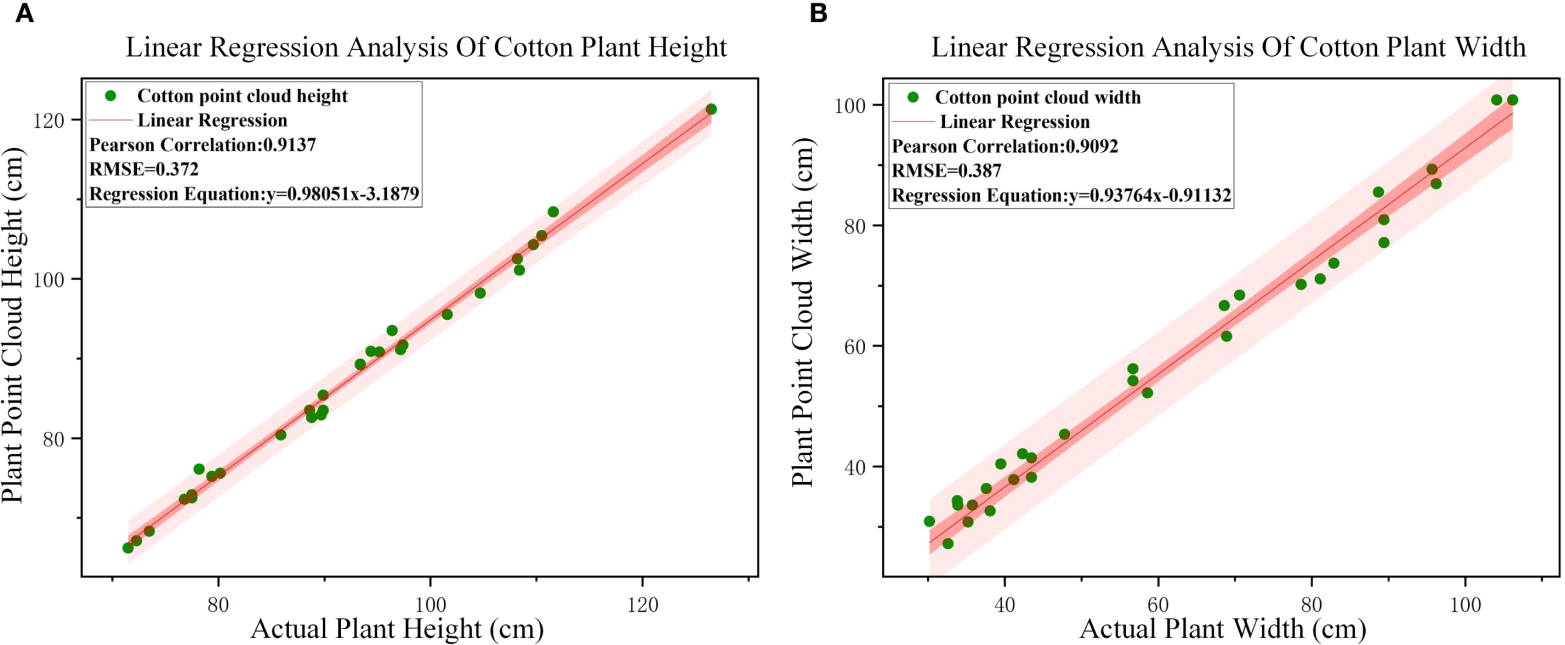
Correlation and RMES analysis results. **(A)** Correlation analysis between calculated and measured value of cotton heights. **(B)** Correlation analysis between calculated and measured value of cotton widths.

The width and height of cotton plants were extracted from the reconstructed point cloud, and the correlation analysis was performed with the measured values in **Table 2**. The error analysis is shown in **Figure 8**. The results showed that the R^2^ value of plant height was 0.9137 and RMSE was 0.372 cm, while the R^2^ value of plant width was 0.9092 and RMSE was 0.387 cm. The R^2^ values of plant height and plant width were all above 0.90, indicating that the proposed plant three-dimensional reconstruction method can accurately realize the construction of complex phenotypic information and has a strong correlation with the actual data.

**Figure 9:**
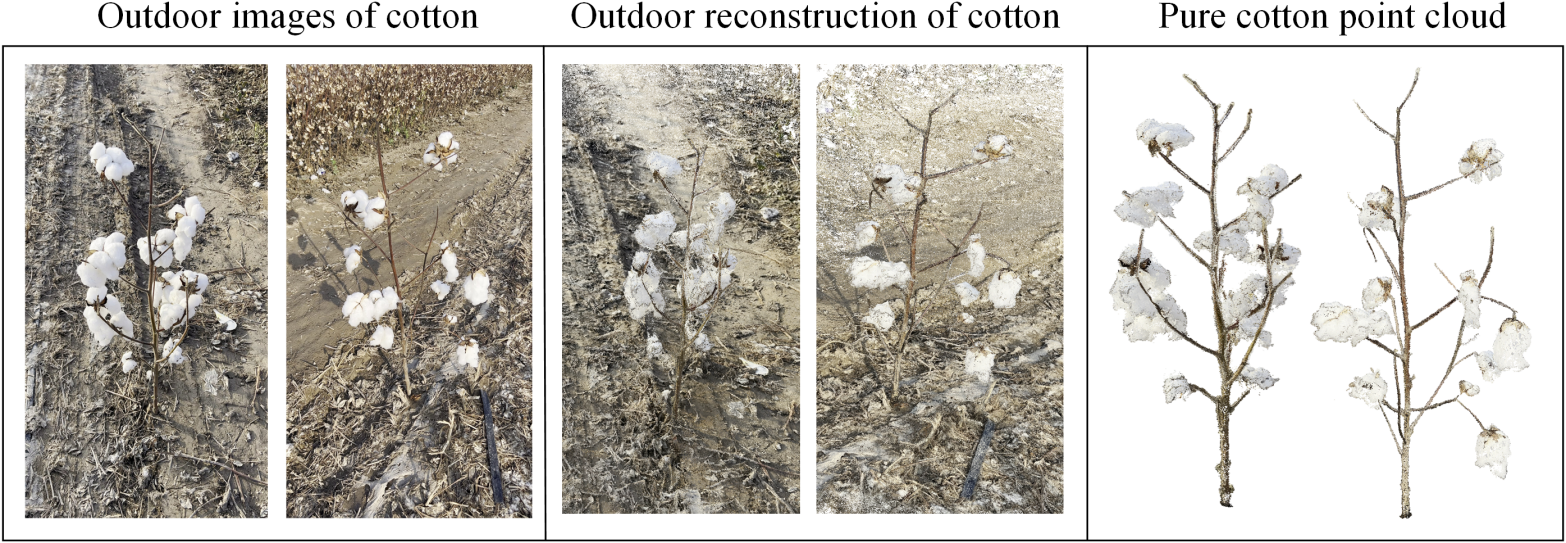
Outdoor cotton data collection and reconstruction results from October 20 to 23, 2024.

In order to evaluate the reconstruction quality under different lighting conditions, this manuscript tests the 3D reconstruction algorithm for different background complexity and different environments (indoor and outdoor). In this manuscript, the data of 10 cotton boll opening periods were collected at Shihezi planting base (outdoor) from October 20 to October 23, 2024 at 19: 00 pm. The collection environment was sunny and the wind level was small and there was no strong light. As shown in **Figure 9**, outdoor data include natural light, drip irrigation tape, plastic film and other complex backgrounds, and three-dimensional reconstruction and reconstruction accuracy verification are carried out. On October 24, 2024, 10 cotton plants were moved into the room, and three-dimensional reconstruction was carried out under a simple indoor background. Regression analysis was performed on the reconstructed cotton plant height and plant width, The RMSE of indoor plant height and plant width after reconstruction were 0.372 cm and 0.387 cm, respectively, and the RMSE of outdoor plant height and plant width were 0.391 cm and 0.402 cm, respectively. The RMSE of plant height and plant width in outdoor were slightly higher than those in indoor by 0.019 cm and 0.015 cm, respectively. It shows that the SFM reconstruction algorithm has good robustness in different environments.

In order to verify the effectiveness of MVS in cotton plant type reconstruction, We used MVS, NeRF and Mip-NeRF methods to reconstruct cotton plants on the same data set. As shown in **Table 4**. Although Mip-NeRF and NeRF methods were slightly higher than MVS in plant height RMSE by 0.076 cm and 0.064 cm, respectively, these two methods significantly increased computer memory consumption and reconstruction time. The MVS method meets the requirements of plant type for plant structure clarity. Considering the reconstruction accuracy and memory consumption, this manuscript uses MVS for three-dimensional reconstruction.

**Table 4:** Comparison of three-dimensional reconstruction methods.

Methods	Plant Height RMSE/cm	Average memory consumption /GB	Average reconstruction time /min
Mip-NeRF	0.300	2.5	170.4
NeRF	0.312	3.6	424.8
MVS	0.376	1.5	40.36

### 3.3 Plant type identification method analysis

#### 3.3.1 Algorithm evaluation

The point cloud data reading, automatic acquisition of multi-view two-dimensional projection and rapid construction of convex hull were carried out on the three-dimensional model of cotton plant after down-sampling. The results are shown in **Table 5**. When the maximum number of point clouds is 75335, the point cloud reading time, multi-view projection time and convex hull construction time are 0.402 S, 2.275 S and 0.018 S, respectively, indicating that the algorithm in this study runs faster.

**Table 5:** Algorithm running time.

Point cloud number	Point cloud reading time/S	Multi-view projection time/S	Convex hull automatic construction time/S
75335	0.402	2.275	0.018
43577	0.147	1.416	0.002

Four experts engaged in breeding have carried out agronomic knowledge in the process of our study. The expert scores are only based on the content of cotton plant type judgment in the ‘Cotton Germplasm Resources Description Specification and Data Standards’, which has certain subjectivity. Therefore, the score of this manuscript is based on the cotton plant type classification described in the ‘Cotton Germplasm Resources Description Specification and Data Standard’ standard and the agronomic knowledge of the experts.

Four cotton agronomists evaluated the proposed plant type identification method from the point cloud reading rate, the automatic acquisition ability of cotton multi-view projection and the automatic construction ability of cotton convex hull in **Table 6** respectively. The average evaluation scores were all above 90, indicating the rationality and innovation of the cotton plant type identification method proposed in this study. Four agronomic experts judged the cotton test set for three times, and used Kappa test to test the consistency of the expert classification results. The Kappa coefficient was calculated to be 0.8655, which was between 0.75-1, indicating that the expert classification results had good reliability.

**Table 6:** Evaluation of cotton plant type identification method.

Evaluation standard	Point cloud reading ability	Cotton multi-view projection automatic acquisition ability	Cotton convex hull automatic construction ability
Scholar 1	90	91	92
Scholar 2	90	93	92
Scholar 3	91	91	90
Scholar 4	92	91	91
Average score	90.75	91.5	91.25

In this study, the data set is increased to 50 cotton plants, of which 20 cotton plants are used as the validation set. The manual measurement method, Canny edge detection, YOLOV10 and the proposed method are compared and analyzed respectively.

(1) Manual measurement method.

Based on the cotton plant type classification described in the ‘Cotton Germplasm Resources Description Specification and Data Standards’ standard and the agronomic knowledge of experts, the cotton plant type were judged as the real plant type label. According to the plant type measurement method specified in the standard of ‘Technical Specification for Identification and Evaluation of Crop Germplasm Resources Cotton’, the length of upper, middle and lower fruiting branches of cotton plants was measured by manual measurement method. If the length of the upper, middle and lower fruit branches of cotton is similar, it is a cylinder type. If the lower fruit branch is long and the upper branch is gradually shortened, it is a tower type. Then the cotton plant type of the verification set is obtained. On the same verification set, the plant type obtained by the cotton plant type judgment method proposed in this study is compared with the results of manual measurement.

(2) Canny edge detection.

In this study, Canny edge detection is used to detect the boundary contours of different cotton plant types on the same verification set. According to the plant type measurement method specified in the standard of ‘Technical Specification for Identification and Evaluation of Crop Germplasm Resources Cotton’, the cross-sectional widths of the upper, middle and lower branches of cotton plants are calculated respectively. If the width values are similar, it is cylinder type, otherwise it is tower type.

(3) YOLOV10.

In this study, YOLOV10 was used to classify cotton plant types, and 200 different plant types of cotton were manually labeled with cylinder and tower types. The training set, test set and verification set are divided into 7: 2: 1 respectively.

In summary, the above three methods are compared with the plant type judgment method proposed in this study. The results are shown in **Table 7**. The Accuracy = 0.75, Precision = 0.80, Recall = 0.72 of this study are higher than the first three methods, indicating that the proposed method reduces the interference of human factors, and there are fewer false detections and missed detections. It can maintain a high ability to distinguish cotton plant types with similar characteristics.

**Table 7:** Comparison of plant type judgment methods.

Method	Accuracy	Precision	Recall
Manual measurement	0.65	0.72	0.67
Canny edge detection	0.40	0.45	0.45
YOLOV10	0.34	0.43	0.38
Our method	0.75	0.80	0.72

#### 3.3.2 Evaluation of plant type threshold

**Figure 10:**
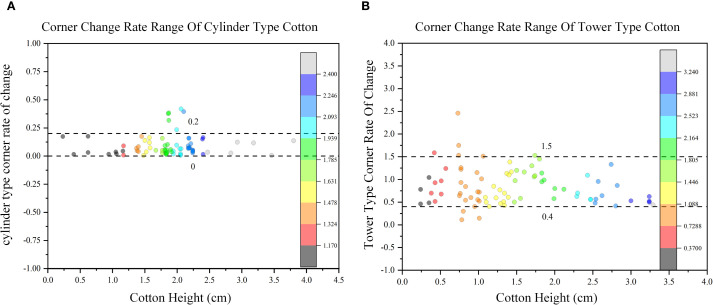
Corner change rate distribution range of different cotton plant types. **(A)** The range distribution of cylinder type. **(B)** The range distribution of tower type.

Firstly, the plant type of all cotton sample data sets were obtained by expert consensus. Based on the plant type given by experts, we project the three-dimensional model into two-dimensional projection, and obtains 1800 two-dimensional projection data of multi-view cotton plants. Then, based on the fast convex hull algorithm, the two-dimensional convex hull of cotton plants under multiple perspectives are constructed, and the range of convex hull corner change rate of all cotton two-dimensional projections are calculated and counted. After counting the corner change rate of different cotton multi-view convex hulls, as shown in **Figure 10**. The conclusion is drawn: **Figure 10A** is the range of cylinder type: 0-0.2, and the distribution range is small for the cylinder type; **Figure 10B** is the range of tower type: 0.4-1.5, and the tower type has a large distribution range.

Twenty cotton plants were used as the validation set. The plant type evaluated by four experts engaged in breeding was used as the real labels, and the corner change rate of cotton in the verification set was calculated and compared with the range of corner change rate obtained in this manuscript. The obtained plant type was used as the prediction labels. The true and predicted plant types are shown in **Table 8**.

**Table 8:** Cotton plant type statistics data.

Variety	Truth plant type	Prediction plant type	Variety	Truth plant type	Prediction plant type
Xinluzao No.40	cylinder type	cylinder type	Xinluzao No.47	tower type	cylinder type
	tower type	tower type		tower type	tower type
Xinluzao No.42	cylinder type	cylinder type	Xinluzao No.48	cylinder type	cylinder type
	cylinder type	tower type		tower type	tower type
Xinluzao No.44	tower type	tower type	Xinluzao No.49	tower type	tower type
	cylinder type	cylinder type		cylinder type	cylinder type
Xinluzao No.45	tower type	cylinder type	Xinluzao No.50	cylinder type	tower type
	tower type	tower type		cylinder type	cylinder type
Xinluzao No.46	cylinder type	tower type	Xinluzao No.52	cylinder type	cylinder type
	tower type	tower type		cylinder type	cylinder type

The cotton plant type data were analyzed, and the results are shown in **Table 9**. The accuracy of cotton plant type Accuracy is 0.75, indicating that the model performs well in the overall classification task. Further analysis of the performance of each category, the precision of the cylinder type and the recall rate of the tower type reached 0.80 and 0.78, respectively, indicating that the model has high reliability in identifying the tower type and the cylinder type.

**Table 9:** Cotton plant type evaluation statistical data.

Plant type	Accuracy	Precision	Recall
Cylinder type	0.75	0.80	0.72
Tower type	0.75	0.70	0.78

#### 3.3.3 Analysis of plant type threshold difference

**Figure 11:**
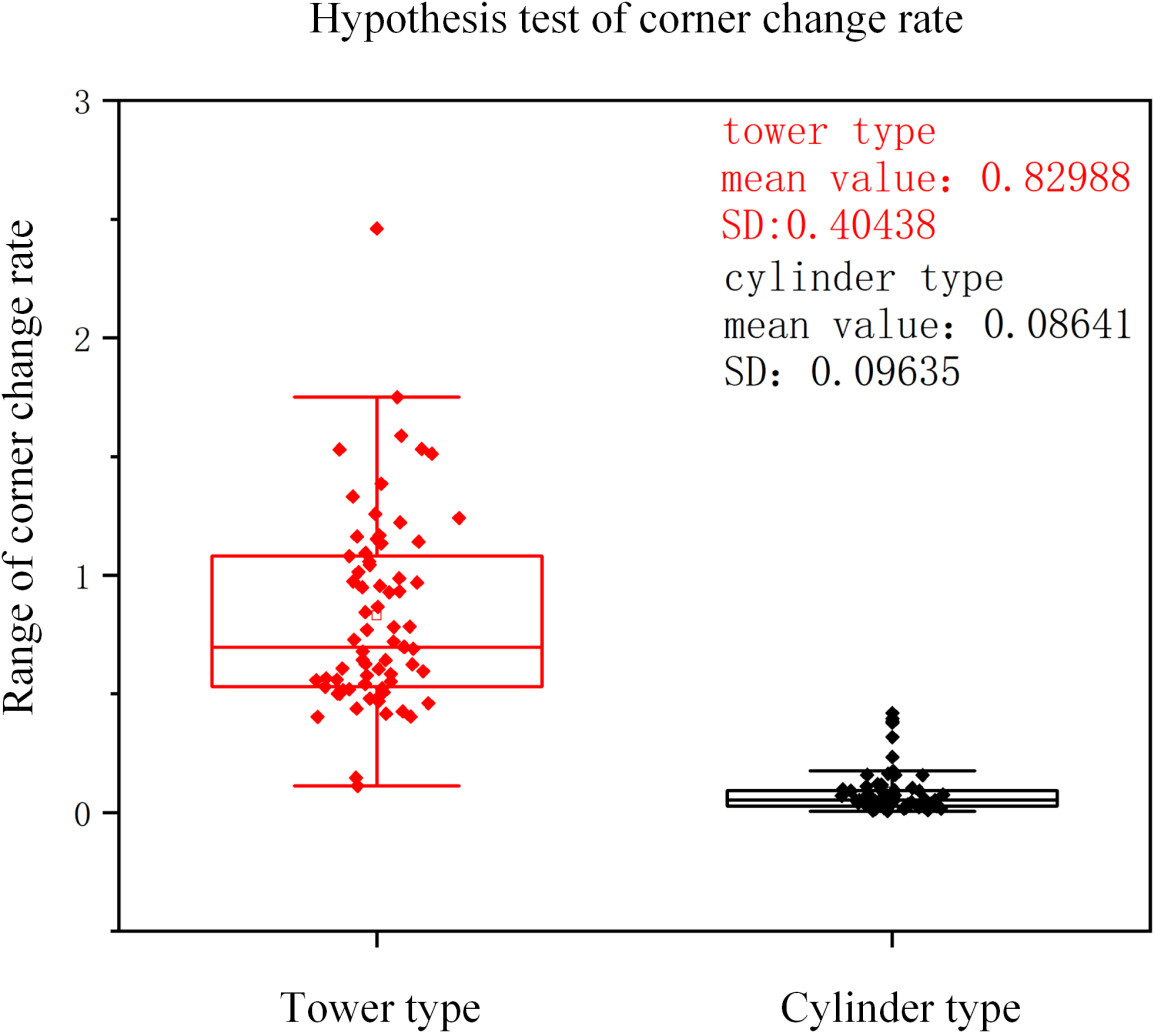
Hypothesis test of corner change rate.

After three-dimensional reconstruction of cotton plants, 1800 two-dimensional images were obtained by two-dimensional projection every 10° interval on all data sets, and the change rate of corner points was calculated respectively. The corner change rate of all cylinder types was calculated, the confidence interval was (0.04687, 0.19357), indicating that most of the sample data were in (0.04687, 0.19357) at 95% confidence level. The corner change rate of all tower types was calculated, and the confidence interval was (0.39987,1.46357), indicating that most of the sample data were (0.39987,1.46357) at 95% confidence. It is basically consistent with the conclusion of this study that the range of cylinder type is 0-0.2, and the range of tower type is 0.4-1.5. The hypothesis test is carried out on the corner change rate data of two different plant types of cotton. The results are shown in **Figure 11**. The sample mean and standard deviation of the tower type were 0.82988 and 0.40438. The sample mean and standard deviation of cylinder type were 0.08641 and 0.09635, respectively. There were significant differences in sample mean and standard deviation between tower type and cylinder type. P = 1.91503×10–^29^ is far less than the commonly used significance level of 0.05, indicating that there is a significant statistical difference between the corner change rate of the tower type and the cylinder type.

In this manuscript, 30 training set samples are subjected to five folds cross-validation. Each fold has six sample points. One fold is taken as the verification set for each experiment, and the remaining four folds are taken as the training set. The corner change rate of cotton samples in the training set was calculated respectively, and the interval range of corner change rate was obtained by statistics. The change rate of the corner points of the samples in the verification set was calculated respectively. Finally, the plant type of the cotton samples in the verification set was obtained by using the interval range obtained from the training set, and compared with the real plant type. Accuracy=0.93, Precision=0.8, Recall=0.9, indicating the stability of the proposed method.

## 4 Discussion

### 4.1 Three-dimensional construction

The data acquisition process in this study is carried out in a windless indoor environment, which avoids the inconsistency of plant images taken from different perspectives due to plant shaking, affects the quality of point cloud reconstruction, and lays a solid foundation for subsequent plant type identification. The incremental SFM algorithm is adopted, compared with the global SFM algorithm, although it is time-consuming and depends on the selection of initial image pairs, the reconstruction accuracy is high and the external points are robust (Gao et al., 2024). Neural Radiance Fields has high quality rendering effect, but it requires long training time and high hardware cost, and its generalization ability is limited (Zhao et al., 2024a). Compared with other three-dimensional imaging methods such as laser radar and laser scanner, the reconstruction algorithm used in this study is lower in cost and meets the needs of plant reconstruction accuracy. It can completely reconstruct the morphological structure of cotton plants and is not destructive to plants (Yu et al., 2024a).

In this study, the three-dimensional point cloud model of cotton plants was successfully constructed in the indoor environment. The R^2^ of the plant height and plant width of the reconstructed three-dimensional point cloud with the measured data was greater than 0.89, and the RMSE was 0.376 cm and 0.395 cm, respectively. Although the accuracy of the reconstructed point cloud meets the requirements of plant type classification, the effects of different lighting conditions, complex background in the field and different sensors on image acquisition and three-dimensional reconstruction are not explored. In the future, different experimental schemes will be designed for discussion.

### 4.2 Plant type identification

In the early stage, some experimental studies were carried out to confirm the projection step size, and the experimental verification was carried out with 5°,10°and15°interval steps. The experimental data are shown in **Table 10**. The results show that the 5° interval can provide more dense sample points, and the calculated corner change rate changes slightly. Compared with this manuscript, the number of projected images increased to 72, and the average multi-view projection time increased to 2.932818/S, which reduced the computational efficiency. The number of two-dimensional images obtained by the 15°interval is too small, the sample points are too sparse and the statistical basis of the data is weak, which cannot fully cover the key boundary area of the convex hull. It will affect the accuracy of the corner change rate distribution. Therefore, in order to improve the computational efficiency and ensure the accuracy of the distribution range of corner change rate, we finally decide to adopt the scheme of projection every 10°.

**Table 10:** Different step size data statistics.

Projection interval /°	Average projected image / frame	Average multi-view projection time /S
5°	72	2.932818
10°	36	1.415909
15°	24	1.020377

In the process of exploring the identification method of cotton plant type, the different viewing angles may affect the morphological structure of cotton plants. In order to reduce the viewing angle error, this study proposes to project the cotton point cloud model to the two-dimensional plane every 10° in the three-dimensional space. In the process of identifying the plant type of cotton plants based on two-dimensional projection, in the early stage, the plant type was judged by analyzing the horizontal cross-sectional width of different heights of plants through the height of cotton plants at the bud stage. However, due to the shelter of leaves on stems and the different heights of cotton plants, there is no uniform height value to traverse all cotton plants and calculate the cross-sectional width. Therefore, this study proposes to use cotton plants at the boll opening stage to avoid the occlusion of leaves on stems. Since the average height value may ignore the cross-sectional characteristics of plants in any case, it is proposed to use convex hulls to capture the contour characteristics of different cotton plants, and then statistically analyze the change rate of convex hull corner points to obtain the identification basis of cotton plant type. When encountering cotton with both cylinder and tower characteristics in the identification process, this study counts the number of points falling into the obtained cylinder and tower ranges respectively. If the number of points falling into the cylinder range is large, it is included in the cylinder type, otherwise the tower type.

In the process of cotton plant type judgment, the three-dimensional model of each cotton plant is projected three times, and three different two-dimensional coordinates are obtained. The average error between all two-dimensional coordinates is calculated to be 5.931×10–^6^ cm, which represents the error of multi-view two-dimensional projection. Three convex hulls are constructed for the two-dimensional image of cotton. The perimeter of the convex hull after three times of construction is calculated, and the maximum perimeter error is 9.119×10–^7^ cm, which represents the error in the construction process of the convex hull. Although the projection error and convex hull construction error are small. However, in the future, it is necessary to optimize the algorithm of two-dimensional projection and convex hull construction to continuously reduce the error.

In this study, the proposed plant type judgment method is compared with other three existing methods. The manual measurement method has high accuracy, but it is time-consuming and labor-intensive, and is easily affected by the operator ‘s subjective judgment and human error. When Canny operator is used to detect unstructured features, it is easy to produce false edges and false detection, thus reducing the accuracy. In the task of cotton plant type classification, whether it is cylinder type or tower type, there are more same characteristics between individuals, and there are relatively few characteristics with obvious discrimination. When using YOLOv10 for plant type classification, it is difficult to effectively identify the key features that distinguish different plant types. In contrast, the method proposed in this study achieves a higher degree of automation, significantly reduces the interference of human factors, and further improves the overall processing efficiency and reliability. The accuracy of this method is 0.75, which is higher than 0.65 of manual measurement and 0.34 of YOLOV10, indicating that this method performs better in correctly classifying cotton plant types.

In terms of three-dimensional reconstruction, real-time reconstruction of cotton plants has not yet been realized. In the future, we will focus on studying more efficient data acquisition methods, reducing reconstruction time and improving reconstruction accuracy. In terms of plant type judgment, the plant type identification method proposed in this study is not applicable to all cotton plants. Only 50 cotton samples are statistically analyzed to provide reference and inspiration for further exploration of cotton plant type identification methods. We acknowledge that the limited cotton data set collected in the experiment limits the universality of the cotton plant type threshold conclusion. In order to further verify the transferability of the conclusion, cotton data was collected every 10 days from the cotton seedling stage in May 2025, a total of 5 varieties were collected, 20 plants per variety, a total of 100 plants. We will collect data from four periods of cotton seedling stage, bud stage, boll stage and boll opening stage. The collection traits included plant height, initial node height of fruit branch, fruit branch angle and so on. The growth status and plant type changes of the whole growth cycle of cotton will be closely monitored to further verify and optimize the conclusions of this manuscript. This study is expected to collect more cotton plants for more detailed statistical analysis of cotton plant type identification basis. In the future, different sensors can be used to collect multi-time series data of cotton, and multi-task joint learning (Sha et al., 2025a) can be introduced to fuse time series data sets of different scales to increase the diversity of data sets and understand the dynamic process of cotton growth more comprehensively. The Siamese neural network (Lu et al., 2024) is introduced to judge the plant type through multi-stage training to improve the accuracy of plant type detection, and further integrate the cotton convex hull automatic construction algorithm into the APP to complete the automatic analysis and identification of cotton plant type.

## 5 Conclusion

The low-cost plant three-dimensional reconstruction technology used in this study can easily reconstruct field plants and describe and analyze the growth of field plants. The R^2^ of the height and width of the plants extracted from the three-dimensional reconstruction model and the manual measurement value is above 0.90, and the RMES is 0.372 cm and 0.387 cm, indicating that the reconstruction method can realize the three-dimensional reconstruction of complex plants. The reconstructed three-dimensional model can better reflect the morphological structure of cotton plants.

By counting the corner change rate of multi-view two-dimensional convex hull of 30 cotton plants, the range of corner change rate of different plant types was obtained: the range of corner change rate was 0-0.2, and the cylinder type has a small distribution range. The range is 0.4-1.5, and the tower type has a large distribution range. When the maximum number of point clouds is 75335, the point cloud reading time, cotton multi-view projection time, and convex hull automatic construction time are 0.402 S, 2.275 S, and 0.018 S, respectively, indicating that the method is fast and efficient. It provides a theoretical and technical basis for cotton mechanical picking and plant type breeding, and also provides an effective reference method for cotton plant type identification.

## Data Availability

The raw data supporting the conclusions of this article will be made available by the authors, without undue reservation.
