# Supplementary material for: Research on cotton plant type identification method based on multidimensional vision
Source: Front Plant Sci. 2025 Oct 13;16:1610577. doi: 10.3389/fpls.2025.1610577 (PMC12554658; doi:10.3389/fpls.2025.1610577)
Supplement: Supplementary file 1 [file Table1.docx]

**3D rotation and 2D projection script :**

filename = 'E:/reconstruction/cotton model/tong/tong-1/tong/tong-subsample6.txt';

data = load(filename);

x = data(:, 1);

y = data(:, 2);

z = data(:, 3);

R = data(:, 4);

G = data(:, 5);

B = data(:, 6);

colors = [R, G, B] / 255;

angle_step = 5;

num_angles = 360 / angle_step;

figure;

h3D = scatter3(x, y, z, 1, colors, 'filled');

xlabel('X');

ylabel('Y');

zlabel('Z');

title('3D Point Cloud');

grid on;

figure;

h2D = scatter([], [], 1, 'k', 'filled');

xlabel('X');

ylabel('Y');

title('2D Projection (xoy Plane)');

grid on;

output_image_folder = 'E:/reconstruction/cotton model/7-8/5-image/';

if ~exist(output_image_folder, 'dir')

mkdir(output_image_folder);

end

for angle = 0:angle_step:350

theta = deg2rad(angle);

rotation_matrix = [cos(theta), 0, sin(theta);

0, 1, 0;

-sin(theta), 0, cos(theta)];

y_shifted = y - 1;

shifted_points = [x, y_shifted, z];

rotated_points = (rotation_matrix * shifted_points')';

x_rot = rotated_points(:, 1);

y_rot = rotated_points(:, 2) + 1;

z_rot = rotated_points(:, 3);

set(h3D, 'XData', x_rot, 'YData', y_rot, 'ZData', z_rot);

title(['3D Point Cloud - Rotation: ', num2str(angle), '°']);

drawnow;

xoy_x = x_rot;

xoy_y = y_rot;

set(h2D, 'XData', xoy_x, 'YData', xoy_y, 'CData', colors);

title(['2D Projection (xoy Plane) - Rotation: ', num2str(angle), '°']);

drawnow;

image_filename = sprintf('%s2D_projection_%03d.png', output_image_folder, angle);

saveas(gcf, image_filename);

output_filename=sprintf('E:/reconstruction/cottonmodel/7-8/2D-/2D_projection_%03d.txt', angle);

output_data_folder = 'E:/reconstruction/cotton model/7-8/2D-/';

if ~exist(output_data_folder, 'dir')

mkdir(output_data_folder);

end

fileID = fopen(output_filename, 'w');

if fileID == -1

error('Failed to open file for writing: %s', output_filename);

end

fprintf(fileID, 'Angle: %d degrees\n', angle);

fprintf(fileID, 'X Y R G B\n');

for i = 1:length(xoy_x)

fprintf(fileID, '%.6f %.6f %.6f %.6f %.6f\n', ...xoy_x(i), xoy_y(i), colors(i, 1), colors(i, 2), colors(i, 3));

end

fclose(fileID);

end

**Convex hull construction script :**

filename = 'E:/reconstruction/cotton model/7-8/2D-modified/2D_projection_005.txt';

data = load(filename);

x = data(:, 1);

y = data(:, 2);

R = data(:, 3);

G = data(:, 4);

B = data(:, 5);

figure;

scatter(x, y, 1, [R, G, B], 'filled');

xlabel('X');

ylabel('Y');

grid on;

hold on;

axis equal;

ylim([0, 6]);

yticks(0:6);

xlim([-4, 4]);

xticks(-4:1:4);

k = convhull(x, y);

convex_hull_vertices = [x(k), y(k)];

plot(x(k), y(k), 'r-', 'LineWidth', 2);

[~, max_index] = max(y(k));

highest_vertex_x = x(k(max_index));

left_points = convex_hull_vertices(convex_hull_vertices(:, 1) <= highest_vertex_x, :);

right_points = convex_hull_vertices(convex_hull_vertices(:, 1) > highest_vertex_x, :);

highest_vertex = [x(k(max_index)), y(k(max_index))];

plot(highest_vertex(1), highest_vertex(2), 'ko', 'MarkerSize', 8, 'MarkerFaceColor', 'k');

marked_left_vertices = [];

marked_right_vertices = [];

for i = 1:length(k)

vertex = convex_hull_vertices(i, :);

if vertex(2) >= 1

plot(vertex(1), vertex(2), 'go', 'MarkerSize', 6, 'MarkerFaceColor', 'g');

text(vertex(1), vertex(2), sprintf('(%0.2f, %0.2f)', vertex(1), vertex(2)), ...

'VerticalAlignment', 'bottom', 'HorizontalAlignment', 'right', 'FontSize', 8);

if vertex(1) <= highest_vertex_x

marked_left_vertices = [marked_left_vertices; vertex];

else

marked_right_vertices = [marked_right_vertices; vertex];

end

end

end

output_dir = 'E:/reconstruction/cotton model/7-8/excel/';

if ~exist(output_dir, 'dir')

mkdir(output_dir);

end

left_output_filename = fullfile(output_dir, 'marked_left_vertices-345.xlsx');

marked_left_vertices = round(marked_left_vertices, 2);

marked_right_vertices = round(marked_right_vertices, 2);

left_table = table(marked_left_vertices(:, 1), marked_left_vertices(:, 2), 'VariableNames', {'X_Left', 'Y_Left'});

writetable(left_table, left_output_filename);

right_table = table(marked_right_vertices(:, 1), marked_right_vertices(:, 2), 'VariableNames', {'X_Right', 'Y_Right'});

writetable(right_table, right_output_filename);

disp;
